# Micro-Electrometric Method for Assessing Cholinesterase Activity in Human Whole Blood

**DOI:** 10.3390/mps8020030

**Published:** 2025-03-11

**Authors:** Nan Ei Moh Moh Kyi, Muhammad Samar, Surat Hongsibsong, Nootchakarn Sawarng, Supansa Pata, Anurak Wongta

**Affiliations:** 1 School of Health Science Research, Research Institute for Health Sciences, Chiang Mai University, Chiang Mai 50200, Thailand; naneimohmoh_kyi@cmu.ac.th (N.E.M.M.K.); muhammad_samar@cmu.ac.th (M.S.); surat.hongsibsong@cmu.ac.th (S.H.); 2 Environmental, Occupational, and NCD Center of Excellent, Research Institute for Health Sciences, Chiang Mai University, Chiang Mai 50200, Thailand; 3 Department of Community Medicine, Faculty of Medicine, Chiang Mai University, Chiang Mai 50200, Thailand; nootchakarn_sawarng@cmu.ac.th; 4 Faculty of Public Health, Chiang Rai Rajabhat University, Chiang Rai 57100, Thailand; 5 Department of Medical Technology, Faculty of Associate Medical Science, Chiang Mai University, Chiang Mai 50200, Thailand; supansa.pata@cmu.ac.th

**Keywords:** cholinesterase activity, pesticide exposure, micro-electrometric method, organophosphorus, carbamate detection

## Abstract

Cholinesterase (ChE) is an essential enzyme for nervous system function, and its activity in the blood serves as a biomarker for pesticide exposure, necessitating accessible assessment methods in resource-limited areas. This study focused on developing and validating a micro-electrometric method to rapidly and reliably measure ChE activity in human blood to monitor pesticide exposure. A micro-electrometric method was designed to evaluate ChE activity in whole-blood samples, involving incubation with organophosphate and carbamate pesticides and subsequent pH measurement, and its performance was compared to that of the Ellman method. The optimized method was strongly correlated with the Ellman method (R^2^ = 0.9147), effectively measuring dose-dependent ChE inhibition by mevinphos and carbofuran with stabilization after 10 min, demonstrating a high sensitivity suitable for field applications. The micro-electrometric method is easy, accurate, and inexpensive for measuring ChE activity. It has been proven to work by comparing it to other methods; therefore, it can be used in clinical and research settings with limited resources.

## 1. Introduction

Cholinesterase (ChE) plays a crucial role in the peripheral and central nervous systems [1]. Determining cholinesterase activity in human blood is an important biomarker of exposure to certain pesticides, such as organophosphorus (OP) and carbamate (CM) [2]. These pesticides are prevalent in pest control; however, their residues present considerable health hazards to humans upon excessive consumption. They stop acetylcholinesterase, which is an important enzyme in the nervous system, from working. Long-term exposure can cause many health problems, including neurological and developmental disorders, cancer, and reproductive disorders [3].

The urgent need for efficient analytical methods to screen OP and CM pesticide residues is critical in developing nations with small-scale agricultural systems. Traditional techniques, such as gas chromatography/mass spectrometry (GC/MS) and liquid chromatography/mass spectrometry (LC/MS), are accurate but impractical because of their complexity and costs. Recent advancements have proposed alternatives for enhancing accessibility and efficiency [4,5,6].

The detection of cholinesterase activity in blood is crucial for assessing toxicity and health status. However, current traditional methods such as the Ellman method have limited sensitivity and specificity due to interference from oxime reactions and hemoglobin absorbance [7]. Various advanced techniques have been developed to address these issues. Reverse-phase HPLC (RP-HPLC) with UV detection enables the quick and accurate measurement of acetylcholinesterase (AChE) activity, especially in populations exposed to pesticides [8]. Surface-enhanced Raman spectroscopy (SERS) can detect butyrylcholinesterase (BChE) activity very well and works well with other spectrophotometric techniques [9]. Electrochemical tests, such as biosensors and ion-selective electrodes, can quickly and accurately measure enzyme activity. These tests are useful for both research and clinical use [10,11,12]. Additionally, innovative methods that utilize electron transfer effects and specialized detection kits have enhanced enzyme stability and sensitivity for practical use [13]. These advancements signify the increasing sophistication of cholinesterase activity detection, providing customized solutions for diverse requirements and contexts.

The increasing demand for monitoring blood ChE activity in pesticide-exposed workers underscores the necessity of a straightforward and economical approach for assessing enzyme activity in red blood cells and serum. This method requires minimal laboratory equipment, circumvents intricate techniques, and facilitates analyses in smaller laboratories. Analytical approaches for measuring ChE activity include titrimetry, pH metrics, and spectrophotometry, using various substrates. Although colorimetric methods provide good accuracy and precision and yield rapid results for plasma ChE, they encounter interference from the glutathione sulfhydryl group and hemoglobin when red blood cells are analyzed [14]. The electrometric method is a quick, easy, and accurate way to check the activity of ChE in the blood, which is important for identifying enzyme inhibition caused by OP and CM pesticide exposure. It is particularly useful in small laboratories because of its minimal equipment requirements and straightforward procedures [15].

However, an earlier modification reported enzyme activity in µmol of acetic acid per milliliter per minute, but this method required expensive and specialized equipment [14]. Another method utilizes automatic titration with standardized sodium hydroxide to monitor acetic acid production; however, this approach also relies on costly and nonstandard laboratory equipment [16]. A simplified electrometric method for assessing human plasma and erythrocyte ChE activities was developed and validated. This method uses a ChE assay with certain amounts of plasma or erythrocytes, distilled water, buffer, and substrate mixed in a controlled environment. Its results have been shown to be the same for all anticoagulants. However, it also lacks biochemical relevance, reporting its activity as Δ pH/20 min [15].

This study aimed to develop a micro-electrometric method that can measure ChE activity, which is expressed as micromoles of acetic acid released per min and is calculated based on the stoichiometry of the reaction and buffer capacity. The timely and accurate detection of potential pesticide exposure should be ensured to monitor ChE activity effectively in various settings.

## 2. Materials and Methods

### 2.1. Materials

#### 2.1.1. Chemicals

Acetylthiocholine iodide (ATCh) and 1,5′ dithiobis (2-nitrobenzoic acid) (DTNB) were purchased from Sigma Aldrich. Standard pesticides including mevinphos and carbofuran were purchased from Dr. Ehrenstorfer GmbH (Augsburg, Germany).

#### 2.1.2. Apparatus

The micro-electrometric measurement method was developed using a portable pH meter (Horiba LAQUAtwin pH-33 Compact Meter Portable pH Meter, HORIBA, Ltd., Kyoto, Japan). The colorimetric method was performed on a UV-vis spectrophotometric microplate reader (Sunrise, Tecan, Männedorf, Zurich, Switzerland).

### 2.2. Specimen Preparation

Blood samples were collected using a finger prick from 30 October to 30 December, 2023. A total of 73 samples were obtained by collecting approximately 150 µL of whole blood from each participant using two heparinized capillary tubes. The blood was gently expelled into the centrifuge tubes. From each participant’s 150 µL sample, 50 µL was pipetted into a pooled blood sample, which was used as the control blood sample for method development. The samples were transported in an ice-cooled container to the Environmental and Occupational Health Science Laboratory at the Research Institute for Health Sciences, Chiang Mai University, and stored at −20 °C until analysis.

### 2.3. Ethical Approval and Consent to Participate

This study was approved by the ethics review board of the Faculty of Associate Medical Technology, Chiang Mai University, prior to data collection (doc no. AMSEC-66EX-062). Consent was obtained from all the participants after they received comprehensive information regarding the study, and their agreement was documented in written form.

### 2.4. Optimization of Micro-Electrometric Method for Whole-Blood ChE Activity

In our study, we employed a revised electrometric technique originally developed by Mohammad et al. [15] and Barenghi et al. [14], which was adapted in our previous study [17] to assess ChE activity levels.

The assay protocol was as follows: 300 µL distilled water (DW), 20 µL of the sample, and 300 µL PBS (pH 8.0) were mixed in a 1.5 mL microcentrifuge tube. ATCh solution (7.5% *w*/*v* in DW) was added to the tube. The initial pH of the mixture (pH1) was measured using a pH meter at 30 s. The mixture was incubated at room temperature (25 °C) for 10 min followed by a second pH measurement (pH2). The pH differences (∆pH) were quantified by calculating the change in pH adjusted for the control tube without a sample, using a defined formula.

∆pH = (pH1 − pH2) − ∆pH of the control

We systematically varied three parameters to optimize the method: ATCh substrate concentration, incubation temperature, and reaction time. For ATCh substrate optimization, volumes of the 7.5% stock solution were tested in the range of 10–40 µL, corresponding to final concentrations of 4.03–16.13 mM. DW was added to each test tube to maintain a constant reaction volume of 630 µL. pH changes were monitored every minute for a total duration of 10 min. The optimal ATCh concentration and reaction time, demonstrating maximum and stable pH changes, were selected for subsequent experiments.

Temperature optimization was performed by measuring the change in pH at 25 °C and 37 °C in a water bath. Using 20 µL of 7.5% ATCh stock solution, measurements were started at the 30 s mark and then at 1 min intervals for 5 min. ∆pH was calculated according to a standard protocol. Temperature conditions that yielded the highest enzyme activity were selected for further experiments, taking into account practical considerations for experimental procedures.

### 2.5. Calculation of ChE Activity of Micro-Electrometric Method

Cholinesterase activity was expressed as U/mL where one unit was defined as the amount of enzyme that catalyzed the conversion of 1 µmol of substrate (released acetic acid) per min under specific conditions, calculated based on the stoichiometry of the reaction and the buffer capacity following a previous study [14].

The number of protons released was calculated using the following equation:

Δ[*H*+] = *β* × ΔpH

where *β* represents the buffer capacity, which is determined by the pH change observed when 10 µL of 0.01 mol/l HCl is added to 600 µL of buffer, using a pH apparatus. The ChE activity in each sample was then calculated using the following equation:

Activity = *d* × [Δ[*H*+]/min]

where *d* is the dilution factor. By combining the above equations, the initial ChE activity could be determined as

Activity = *d* × *β* × [ΔpH/min]

### 2.6. Colorimetric Method for Measurement of ChE Activity

ChE activity was determined following our previously described method developed from the original Ellman method [18,19]. Whole-blood samples (10 µL) were diluted 1:300 in PBS (pH 7.4). Subsequently, 50 µL of each diluted sample was mixed with 200 µL of a 1:1 mixture of ATCh and DTNB in PBS in a 96-well microplate. Absorbance was measured using a kinetic method with a UV-vis spectrophotometric microplate reader. The initial background absorbance was recorded at 405 nm, followed by a 5 min reaction period. Enzyme activities were calculated by multiplying the difference between the initial and 5 min absorbance values by the blood reaction factor, and the results were expressed in U/mL, where one unit was defined as the amount of enzyme which catalyzed the conversion of 1 µmole (micromole) of substrate per min under specific conditions.

### 2.7. Blood ChE Inhibition by Organophosphate and Carbamate Pesticides

A preliminary assay was performed to determine the optimal duration for a complete reaction between ChE and the inhibitor. Briefly, 20 µL of control whole blood was added to a tube, followed by 20 µL of 5 ppm mevinphos dissolved in 5% methanol in PBS (pH 8.0), resulting in a final concentration of 2.5 ppm. The mixture was then incubated at 25 °C for varying durations ranging from 5 to 20 min. After incubation, 590 µL of a 1:1 (*v*/*v*) mixture of distilled water and PBS was added to each tube. ChE activity was measured at 25 °C for 5 min, as described above, using the micro-electrometric method. The optimal inhibition time determined from these trials was used in subsequent experiments.

For the main assay, 20 µL of control whole blood was added into a tube, followed by 20 µL of OP (mevinphos) and CM (carbofuran) inhibitors at varying concentrations (0.1, 0.5, and 1.0 ppm) dissolved in 5% methanol–PBS (pH 8.0), resulting in final inhibitor concentrations of 0.05, 0.25, and 0.5 ppm. The solution was then incubated for 10 min. ChE activity was assessed at 25 °C for 5 min using the methodology outlined in the previous section.

The inhibition rate was calculated using the following equation:

%Inhibition = (ChE control − ChE inhibited)/ChE control × 100

where [ChE control] is the initial enzyme activity in the absence of an inhibitor, and [ChE inhibited] is the enzyme activity in the presence of an inhibitor.

### 2.8. Statistical Analysis

The ChE activity findings were reported as mean values and standard deviations. Variations in ChE activity during the study were assessed via t-tests and one-way ANOVA. All statistical analyses were conducted as two-sided tests with a significance threshold set at 0.05.

## 3. Results

**Figure 1:**
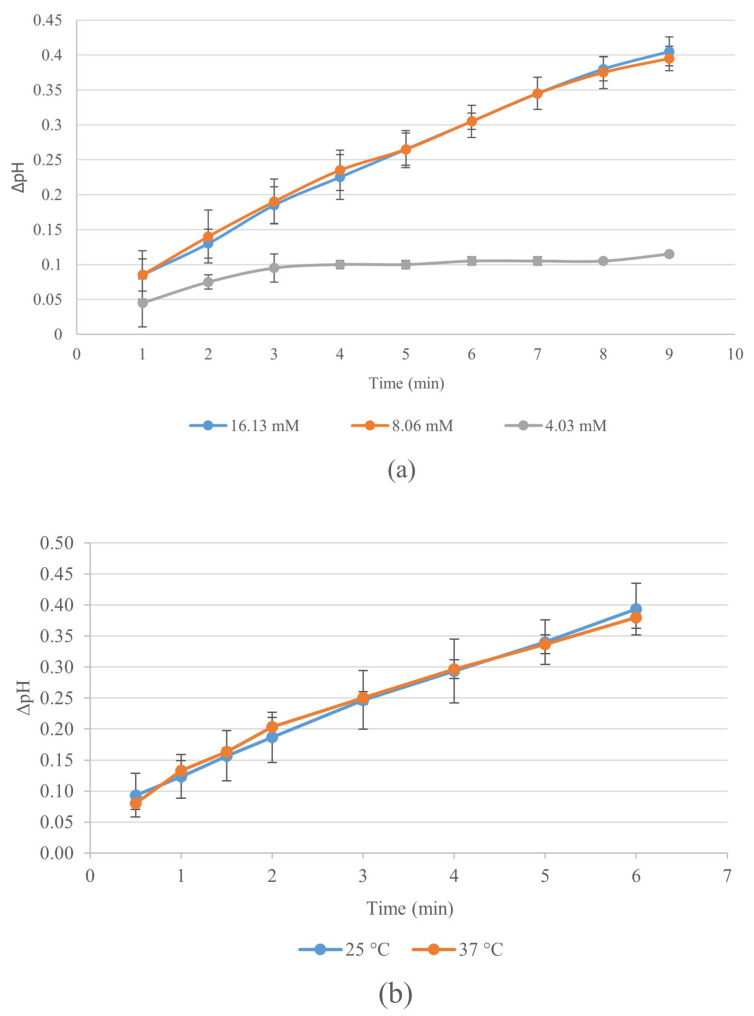
Effect of ATCh concentration (**a**) and temperature (**b**) on ΔpH in the developed micro-electrometric method. (**a**) ΔpH was measured at different ATCh concentrations (4.03, 8.06, and 16.13 mM) at 25 °C over 10 min. (**b**) Temperature optimization was performed using an ATCh concentration of 8.06 mM, comparing reaction conditions at 25 °C and 37 °C.

To optimize the micro-electrometric method, we assessed the influence of ATCh substrate concentration and temperature on ChE activity. Three ATCh concentrations (4.03 mM, 8.06 mM, and 16.13 mM) were evaluated at 25 °C, with ΔpH monitored over 10 min. Measurements were performed in duplicate, with standard deviation represented by error bars. The optimization results are shown in Figure 1a. Both 8.06 mM and 16.13 mM yielded the highest ΔpH values without significant differences due to overlapping error margins, while 4.03 mM exhibited notably lower activity. Therefore, 8.06 mM was designated as the optimal concentration for balancing performance and cost-effectiveness. At 4.03 mM, smaller error bars at extended time intervals likely resulted from substrate depletion, diminishing enzyme activity and variability. Moreover, the decision to conduct duplicate instead of triplicate measurements may have led to an underestimation of variability. Conversely, higher ATCh concentrations (16.13 mM) resulted in increased enzyme activity, which contributed to greater variability in reaction kinetics and pH shifts.

A 5 min incubation period was deemed optimal, as most pH changes occurred within this timeframe, ensuring a stable and maximal ΔpH for accurate enzyme activity evaluation. Although the pH continued to rise slightly until 9 min, the additional change was minimal and did not significantly improve assay sensitivity. Extending the incubation time could have introduced external variability (e.g., temperature fluctuations, evaporation), impairing reproducibility. Thus, a 5 min reaction time guaranteed efficient, reliable, and practical detection, suitable for both laboratory and field settings.

For temperature optimization (Figure 1b), ΔpH was assessed at 25 °C and 37 °C using the 8.06 mM ATCh concentration. Both temperatures exhibited a consistent increase in ΔpH, yet significant overlap in error margins indicated no substantial difference in ChE activity under the conditions tested. Given this similarity, 25 °C was selected as the optimal temperature for its practicality in field applications, negating the necessity for external temperature regulation.

**Figure 2:**
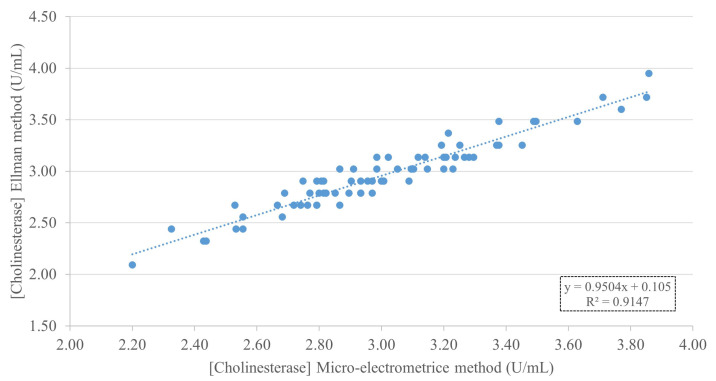
Correlation between ChE activity measured using the developed micro-electrometric method and the Ellman method.

Seventy-three whole-blood samples were tested and compared using the standard method. The developed micro-electrometric and Ellman methods were used to analyze and calculate the correlation. The results are shown in Figure 2, which shows a strong positive correlation between the ChE activity measured using the developed micro-electrometric method and the Ellman method. The equation y = 0.9504x + 0.105 represents a linear regression line with a value of R^2^ 0.9147. This high degree of correlation suggests that the micro-electrometric method is consistent with the Ellman method for measuring ChE activity.

The percentage inhibition of ChE activity in control whole-blood samples was measured at different incubation times (5, 10, 15, and 20 min) with 5 ppm mevinphos, which is shown in Table 1. Measurements were conducted in triplicate, and the error values indicated standard deviation. The results indicated that the inhibition of ChE activity increased with the incubation time. At 5 min, the percentage of inhibition was 87.5% (±2.4), which significantly increased to 95.8% (±4.2) at 10 min. Inhibition remained steady at 95.8% (±4.2) after 15 min and further increased slightly to 97.2% (±2.4) after 20 min, though this difference was within the measurement error. The consistent inhibition observed at 10, 15, and 20 min (denoted by “a”) suggests that enzyme inhibition reached a plateau after 10 min of incubation.

Inhibition was performed for 10 min, and the results are presented in Table 2. Both mevinphos and carbofuran significantly inhibited ChE activity in a dose-dependent manner. At 0.50 ppm, mevinphos and carbofuran reduced ChE activity by 69% and 60%, respectively. Lower concentrations of both pesticides showed decreased inhibition, with mevinphos and carbofuran at 0.25 ppm resulting in 60% and 56% inhibition and at 0.05 ppm in 40% and 44% inhibition, respectively. These effects were statistically significant in decreasing ChE activity from the baseline (*p* < 0.05).

## 4. Discussion

In developing the micro-electrometric method for whole-blood cholinesterase detection in our study, several key factors were optimized to ensure the accurate and reliable measurement of ChE activity in human blood. The developed method can be carried out at room temperature, which means that it can be widely used in the field, similar to previous ChE screening methods [20,21]. Correlation analysis between the micro-electrometric and established Ellman methods demonstrated a strong positive correlation. This finding confirms that the micro-electrometric method is consistent with the Ellman method, which is consistent with another previous method [14], providing a reliable alternative for measuring ChE activity. The strong correlation of the micro-electrometric method suggests that it can be used confidently instead of more complex and expensive techniques, particularly in settings with limited resources.

Traditional electrometric methods for cholinesterase analysis often express enzyme activity as ΔpH per min or hour but lack biochemical significance. To address this, a modified Michel’s method was introduced in a previous study, quantifying the results in µmol of acetic acid per milliliter per min. However, this method required costly specialized equipment. Our method is biochemically significant because it directly measures proton release from acetic acid production, a key product of cholinesterase-mediated hydrolysis. Unlike previous electrometric approaches which merely track pH changes without linking them to enzymatic activity, our method quantifies ChE function in meaningful biochemical terms. However, our study did not directly assess substrate specificity using a non-ChE substrate as a negative control. Future experiments should include such controls to confirm that the observed pH changes are exclusively due to cholinesterase activity and not other physicochemical interactions. Additionally, investigating whether inhibitors such as mevinphos and carbofuran have any non-specific effects on pH change in the absence of ChE activity would further validate the biochemical specificity of our approach. Recent developments have led to a simplified electrometric technique that utilizes affordable and user-friendly equipment, enabling accurate ChE activity calculations without expensive equipment. This new method demonstrated comparable sensitivity and reliability, making it accessible for broader application in both clinical and research settings [14,22,23,24,25].

This study also evaluated the percentage inhibition of ChE activity at different incubation times, and the results showed a significant increase in inhibition, from 87.5% at 5 min to 95.8% at 10 min, which stabilized thereafter. This stabilization suggests that a 10 min incubation is sufficient for achieving maximal inhibition, optimizing the efficiency of the method without compromising accuracy. The in vitro inhibition of ChE activity by mevinphos and carbofuran in our study aligns with established findings regarding the anti-ChE effects of these insecticides. Studies indicate that OP, such as chlorpyrifos, significantly reduce ChE activity, with evidence showing that exposure leads to concentration-dependent cytotoxicity in various biological samples [26]. In vitro carbaryl assays demonstrated the concentration-dependent inhibition of blood AChE and plasma BChE, which highlights the sensitivity of these assays in detecting alterations in enzyme activity [27]. The developed methods employed in these studies demonstrated sensitivity in detecting ChE inhibition, supporting the notion that OPs and CMs pose a risk to ChE function. Pesticide-induced ChE inhibition varies with exposure level and duration, with inhibition greater than 70% indicating severe toxicity and clinical symptoms. The ability of our method to detect the dose-dependent inhibition of ChE activity by mevinphos and carbofuran highlights its potential as a practical tool for monitoring individuals at risk of pesticide exposure [28,29]. These findings underscore the importance of monitoring pesticide exposure and its potential neurotoxic effects on human health.

In summary, new techniques such as RP-HPLC and SERS improve sensitivity but often require costly specialized equipment [8,9]. Electrochemical methods, such as biosensors, can offer rapid measurements, but their complexity may hinder their widespread adoption [10,11,12]. Conversely, our micro-electrometric method provided similar sensitivity and reliability to the traditional Ellman method. However, the Ellman method has limitations in sensitivity and specificity due to interference from hemoglobin. Our micro-electrometric method addresses this challenge by directly measuring pH change, eliminating reliance on colorimetric detection, and reducing interference from hemoglobin in whole-blood samples. However, we acknowledge that hemoglobin interference was not explicitly tested in this study. Future investigations should assess potential hemoglobin effects to further validate the method’s robustness in whole-blood samples. These benefits highlight the potential of our method to address the significant gaps in ChE activity monitoring, especially in resource-limited settings, making it a valuable tool for monitoring pesticide exposure and its effects on human health.

## Figures and Tables

**Table 1:** Effect of incubation time and 5 ppm mevinphos on the percentage inhibition of ChE activity using the micro-electrometric method.

	Incubation Time (Minutes)			
	5	10	15	20
% Inhibition (Mean ± SD)	86.1 ± 2.4	95.8 ± 4.2 ^a^	95.8 ± 4.2 ^a^	97.2 ± 2.4 ^a^

Abbreviations: ^a^ means followed by the same letter are not significantly different (*p* > 0.05).

**Table 2:** Effect of pesticide concentration on ChE activity and percentage inhibition.

Insecticide Conc. (ppm)	ChE Activity (U/mL)	Inhibition (%)
Baseline 0.0	(2.87 ± 0.07)	
Mevinphos		
0.5	(0.89 ± 0.13) *	69
0.25	(1.14 ± 0.34) *	60
0.05	(1.73 ± 0.26) *	40
Carbofuran		
0.5	(1.14 ± 0.13) *	60
0.25	(1.27 ± 0.25) *	56
0.05	(1.61 ± 0.15) *	44

Abbreviations: * significantly different at *p* < 0.05.

## Data Availability

Data are contained within the article.
